# Application of *Cyclocarya paliurus*–Kiwifruit Composite Fermented to Enhance Antioxidant Capacity, Flavor, and Sensory Characteristics of Kiwi Wine

**DOI:** 10.3390/molecules29010032

**Published:** 2023-12-20

**Authors:** Jing Liu, Weiliang Guan, Zhidong Sun, Yunfan Ni, Long He, Fang Tian, Luyun Cai

**Affiliations:** 1 Key Laboratory of Health Risk Factors for Seafood of Zhejiang Province, College of Food Science and Pharmaceutics, Zhejiang Ocean University, Zhoushan 316000, China; lj15385454539@163.com (J.L.); n1245949415@163.com (Y.N.); hl18868018290@163.com (L.H.); 2 School of Biological and Chemical Engineering, NingboTech University, Ningbo 315000, China; wlguan@zju.edu.cn (W.G.); zdsun_cn@163.com (Z.S.); 3 Ningbo Innovation Center, College of Biosystems Engineering and Food Science, Zhejiang University, Ningbo 315100, China

**Keywords:** *Cyclocarya paliurus*, kiwi wine, undersized kiwifruits, flavor compounds, antioxidant capacity, sensory analysis

## Abstract

A new fermentation method for kiwi wine was explored by developing the well-known medicinal and edible plant *Cyclocarya paliurus* (*C. paliurus*) to create more value with undersized kiwifruits. In this study, the changes in bioactive substances during the *C. paliurus*–kiwi winemaking process were analyzed on the basis of response surface optimization results, and the antioxidant capacity, aromatic compounds, and sensory quality of the *C. paliurus*–kiwi composite wine with kiwi wine and two commercial kiwi wines were compared. The results showed that DPPH radical, OH^−^ radical, and ABTS^+^ scavenging rates remained at over 60.0%, 90.0%, and 70.0% in *C. paliurus*–kiwi wine, respectively. The total flavonoid content (TFC) and total polyphenol content (TPC) of *C. paliurus*–kiwi wine were significantly higher than those of the other three kiwi wines. *C. paliurus–kiwi* wine received the highest score and detected 43 volatile compounds. Ethyl hexanoate, which showed stronger fruity and sweet aromas, was one of the main aroma components of *C. paliurus*–kiwi wine and different from commercial wines. This wine has a good flavor with a natural and quality feeling of *C. paliurus*–kiwifruit extract, low-cost processing, and great market potential.

## 1. Introduction

*Cyclocarya paliurus* (Batal) Iljinskaja (*C. paliurus*) is a traditional, medicinal, and edible plant distributed in southern China [1]. In past studies, the main focus has been on the activity studies and cytotoxic assays of *C. paliurus* extracts and compounds. Its leaves have been widely used as a remedy for hyperlipidemia in traditional medicine. Xie et al. [2] demonstrated that its leaves are commonly used as a remedy for hyperlipidemia. *C. paliurus* leaves ethanol extract, which shows the potential to clean hepatic fat and may be significant in the treatment of diabetic cardiomyopathy [3,4]. *C. paliurus* polysaccharide can also treat type 2 diabetes mellitus [5]. Furthermore, *C. paliurus* can be added to functional foods and dietary supplements. At present, *C. paliurus* leaves are utilized as green vegetables or made into tea, which is a traditional, healthy, sweet herbal beverage in China [6,7]. Other beverage products of *C. paliurus*, such as alcoholic products, are yet to be developed.

Kiwifruit (*Actinidia Lindl*) is a fruit with a sweet taste and is rich in vitamin C, various essential amino acids, and other nutrients [8]. Currently, China produces more than half the world’s kiwifruit. However, the increase in production has also produced a large number of perishable, undersized kiwifruits, which are not suitable for sale [9]. Kiwifruit growers are looking to manufacture other value-added products from undersized kiwifruit. Therefore, the kiwifruit industry needs to diversify its products, such as kiwi wine. Processing defective fruits into value-added food products such as fermented beverages could reduce orchard economic losses and maximize the use of fruit resources [10].

In fruit wines and beverages, fruity aroma is an important flavor profile and the main distinguishing characteristic as it embodies sensory pleasure [11]. Since the kiwi wine aroma is not attractive enough, many attempts have been made to improve it, such as mixed fermentation with compound yeasts, fermentation with fruit peel, or selection of kiwifruit varieties, etc. Mixed fermentation produces more popular fragrances, such as the flower and sweet fruit notes of wines, and produces more volatile compounds compared with pure fermentation [12]. Kiwifruit peel is a potentially valuable source of compounds and has a higher antioxidant level than fruit pulp [13]. Fermentation with peel and pulp can increase the aromatic content of fruit wine. Tea is considered a suitable medium for fermentation and can improve the quality of wine. For example, CTC (crush, tear, and curl) tea is typical for producing tea cider [14]. *C. paliurus* leaves are also a kind of traditional Chinese tea. Likewise, the *C. paliurus* aqueous extracts show a bright brownish-yellow color, which makes the wine sample more translucent. The sales of easy-drinking wine with medium alcohol content have been growing in Europe [15]. Proper drinking of fruit wine, including medium alcohol content fruit wine, is beneficial to human health [16]. Therefore, we proposed the combination of *C. paliurus* and kiwifruit to produce a kiwifruit–tea wine with medium alcohol content and to improve the nutrient value and enhance the aroma of kiwi wine [17]. In addition, this proposal aimed to reduce the waste of undersized kiwifruit and improve the income of fruit farmers.

In this research, we analyzed the antioxidant levels and their changes during the *C. paliurus*-blended kiwi wine (herein called *C. paliurus*–kiwi wine) making process. We also explored alterations in physicochemical properties and the correlation of physicochemical properties with antioxidant activity. At the same time, we also compared *C. paliurus*–kiwi wine, kiwi wine, and two commercial kiwi wines in order to have a more comprehensive understanding of the similarities and differences in the antioxidant capacity and aromas of *Cyclocarya paliurus*–kiwifruit composite fermented wine and kiwi wine. Undersized “Xuxiang” kiwifruits were chosen as the main components to make *C. paliurus*–kiwi wine. The making process of *C. paliurus*–kiwi wine was refined into an alcoholic fermentation and resting process, making the whole process more systematic. The *C. paliurus*–kiwi wine opens up a new direction for the development of *C. paliurus*, which is only available as a tea drink currently. We hope that the results will provide a theoretical foundation for *C. paliurus*–kiwi winemaking.

## 2. Results and Discussion

### 2.1. Nutrient and Antioxidant Capacity

RSM is a statistical method to optimize projects and improve experimental efficiency. This method has been applied in the wine fermentation of fruits such as cactus pear, mango, and date palm in early reports [18,19,20]. The optimum *C. paliurus*–kiwi winemaking condition (data provided in the supplementary material) was optimized by RSM, and alterations in the physicochemical properties and antioxidant activity of wine were analyzed according to this condition. The TFC, TPC, and other substances in *C. paliurus* and kiwifruit could effectively scavenge free radicals. Martínez et al. [21] used more than one method in order to gain a fuller picture of the antioxidant capacity of fruit co-products. The total antioxidant activity of the sample was evaluated using four indicators (DPPH, ABTS^+^, OH^−^, and O_2_^−^ scavenging activity). Then, the correlations among time, physicochemical properties (TFC, TPC, and ABV), and antioxidant capacity (DPPH, ABTS^+^, OH^−^, and O_2_^−^) of *C. paliurus*–kiwi wine were analyzed.

**Figure 1:**
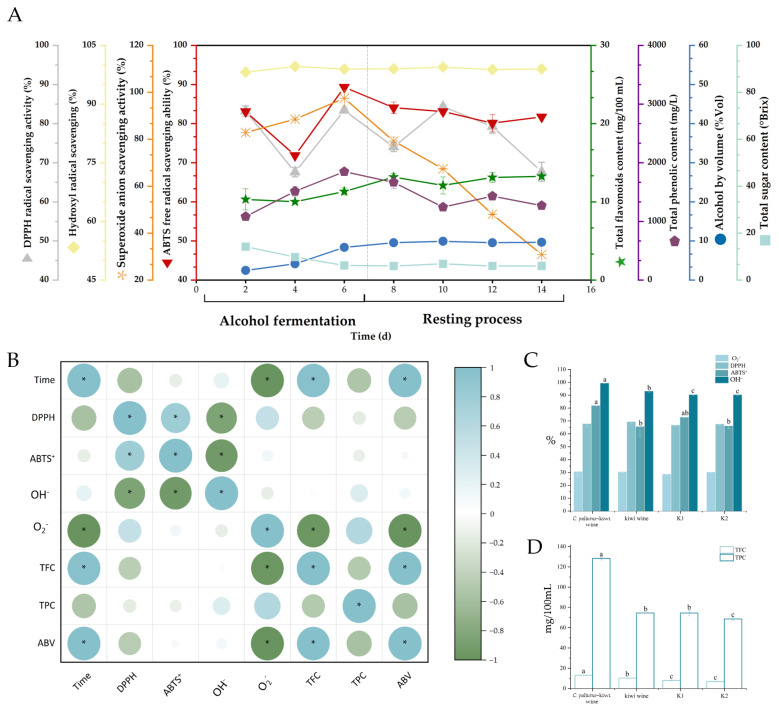
(**A**) Changes in TFC, TPC, ABV, and antioxidant capacity during the winemaking process of *C. paliurus*–kiwi wine (the same below). (**B**) Correlation analysis among TFC, TPC, ABV, and antioxidant capacity. (**C**) Comparison of antioxidant capacity in four kiwi wines. (**D**) Comparison of TFC and TPC contents in four kiwi wines. Asterisks * and different letters represent significant differences (*p* < 0.05).

Figure 1A showed that the DPPH radical scavenging ability fluctuated in the first 10 days of the winemaking process and then fell slowly but remained above 60.0% throughout the 14 days of the winemaking process of the *C. paliurus*−kiwi wine. The study of wines made from eight muscadine varieties showed that “Noble” among them had the same variation as *C. paliurus*–kiwi wine [22]. The same DPPH radical scavenging capacity variation was found in grenadine pomegranate wine by Xie et al., and Lan et al. [2,23] proved that the DPPH radical scavenging ability of flavonoids from *C. paliurus* was dose-dependent and was 38.3% to 94.7% for the concentrations of flavonoids at 10 to 80 mg/100 mL. Wang et al. [24] speculated that the change in polyphenol content was responsible for the change in DPPH scavenging capacity. ABTS^+^ scavenging activity decreased to 71.9% initially, then increased during the 4th to 6th days of fermentation, and remained relatively stable at over 80.0% in the 6th to 14th days of the *C. paliurus*–kiwi winemaking process. ABTS^+^ and FRAP showed the same decreasing trend in the white wine, as antagonistic interactions between polyphenols may occur [24]. These changes in the fermentation process are due to the interactions of microorganisms and enzymes, resulting in the decomposition and transformation of substances [25]. The O_2_^−^ scavenging activity went up and then fell sharply. Lei et al. [26] demonstrated that ABTS^+^ and O_2_^−^ scavenging activities were 18.0–63.2% and 35.6–61.6% at 5.00–25.0 mg/100 mL of *C. paliurus* flavonoids extraction in liquid form. However, a slight decrease in O_2_^−^ scavenging activity was observed in pomegranate wine. After fermentation, the antioxidant activity in orange wine will be lower than that in orange juice [27]. Different varieties of wine showed different patterns of change in O_2_^−^ scavenging activity during fermentation, reflecting the differences in their phenolic composition. The OH^−^ radical is a reactive oxygen species formed in vivo that can cause serious damage to biomolecules [28]. The OH^−^ radical scavenging ability in the *C. paliurus*–kiwi wine samples was strong and remained unchanged.

TPC increased in *C. paliurus*–kiwi wine made with “Xuxiang” kiwifruit during early fermentation and later stabilized after yeast fermentation, as shown in Figure 1A. Earlier research found that the TPCs in “Hayward” and “Guichang” wines considerably increased, whereas those in “Jinkui”, “Milian One”, and “Yate” wines showed a decreasing trend after fermentation [29]. Changes in the TPC of *C. paliurus*–kiwi wine may be related to bioconversion of the non-free forms of phenolic compounds and the metabolism of yeast in the fermentation broth. TFC in wine increased during the winemaking process. The same result was found in other research [30,31]. The increase may be due to the fact that flavonol compounds have simpler structures; polyphenols were broken down and converted into flavonoids by enzymes during the winemaking process of *C. paliurus*–kiwi wine [32]. As shown in Figure 1B, ABV (r = 0.857) and TFC (r = 0.885) were significantly and positively correlated with time. A negative correlation was found between O_2_^−^ scavenging activity (r = −0.870) and time. Additionally, ABV was found to be highly and positively correlated with TFC (r = 0.884). The total sugar content of *C. paliurus*–kiwi wine decreased continuously and sharply from 18 to 6 °Brix during 7 days of the alcohol fermentation. At the end of alcohol fermentation, the alcohol content was 9.60 vol.%. The addition of sucrose is an important way to increase the ABV of *C. paliurus*–kiwi wine [33]. During the resting process, the concentration of alcohol content remained the same. Figure 1C,D showed that the O_2_^−^ scavenging activity, TFC, and TPC of *C. paliurus*–kiwi wine were significantly higher than those of the other three kiwi wines. This phenomenon is closely related to the abundance of organic compounds, especially flavonoids, in *C. paliurus* leaves [2].

In conclusion, the TFC content in the wine gradually increased as the alcohol content increased. The antioxidant activity during the winemaking process remained at a relatively high and stable level. The TFC and TPC of *C. paliurus*–kiwi wine were higher than the other three kiwi wines. Thus, the effective antioxidant capacities of *C. paliurus*–kiwi wine might improve some nutritional principles.

### 2.2. Volatile Aroma Compounds in Four Kiwi Wines

**Figure 2:**
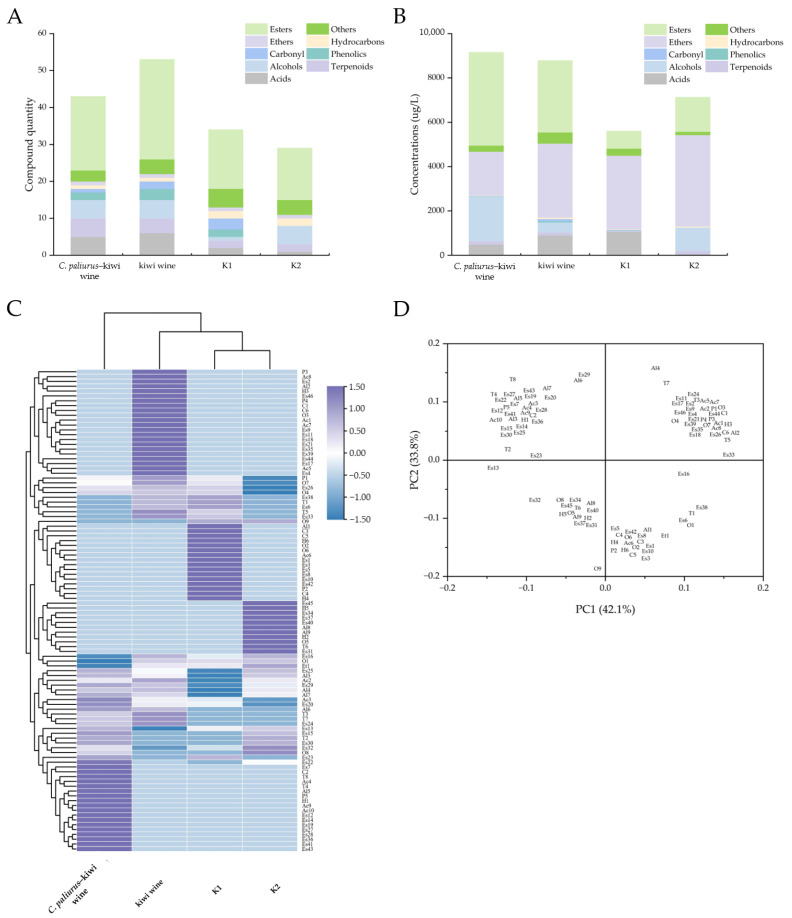
(**A**) Comparison of volatile compound quantities in four kiwi wines. (**B**) Comparison of volatile compound contents in four kiwi wines. (**C**) Heat map of volatile aroma substances in four kiwi wines. (**D**) Principal component analysis (PCA) for 100 volatile flavor compounds. The codes for volatile flavor compounds were defined in Supplementary Table S3.

Wine flavor comes from the mutual coordination of esters, alcohols, acids, aldehydes, ketones, and other compounds. Other factors, such as ethanol content and the use of pectinase, are also important for the aroma characterization of kiwi wine. Ethanol can influence the release of volatile compounds [34]. Pectinase increases the juice yield of kiwifruit and contributes to the clarification of fruit wines. Jiang et al. [35] demonstrated that the content of aromatic compounds such as higher alcohols, esters, and terpenes increased significantly in dragon fruit wine with pectinase pre-treatment. During the resting process, the content of various volatile components, such as ethanol, obutanol, isoamyl alcohol, caprylic acid, and capric acid, tends to decrease and eventually stabilize. The main aroma components of four kiwi wines are formed during the alcohol fermentation stage. As shown in Figure 2A,B, 43 volatile compounds were detected in *C. paliurus*–kiwi wine, totaling 9148.92 μg/L. kiwi wine, K1, and K2 contained 53, 34, and 29 flavor substances, with mass concentrations of 8783.47, 5604.15, and 7126.43 μg/L, respectively. The *C. paliurus*–kiwi wine contained slightly fewer types of volatile compounds but had higher mass concentrations of volatile compounds than kiwi wine, with differences in esters (4193.83 μg/L vs. 3236.88 μg/L) and alcohols (2012.47 μg/L vs. 451.810 μg/L).

These volatiles were classified into eight categories, namely esters (46 types), acids (10 types), alcohols (9 types), terpenoids (8 types), carbonyl (6 types), hydrocarbons (6 types), phenolics (5 types), ether (1 type), and others (9 types). A total of 100 substances were detected in four kiwi wines. Esters impart sweet and fruity odors and occupy an important part of the alcoholic fermentation by-products of the *C. paliurus*–kiwi wine [36]. They are derived from the fermentation products of brewer’s yeast and the enzymatic esterification of higher alcohols and fatty acids. The esters dominated in terms of number. The number of esters in *C. paliurus*–kiwi wine, kiwi wine, K1, and K2 were 20, 27, 16, and 14, respectively. Additionally, the *C. paliurus*–kiwi wine had the highest ester mass concentration among the four wines, reaching 45.8%. The content of volatile acids in *C. paliurus*–kiwi wine is greatly increased by fermentation. Acids balance the fruit aromas in wine. Acetic acid and octanoic acid are high in the four types of wine [37]. Acetic acid makes the wine sweet and cheesy. Among the alcohols, 3-methyl-1-butanol and phenylethyl alcohol in the *C. paliurus*–kiwi wine (1323.32 and 572.120 μg/L) were much higher than those in the kiwi wine (176.050 and 181.040 μg/L) and K2 (28.3900 and 84.4500 μg/L) and were not detected in K1. 3-methyl-1-butanol has a whiskey, malt, and burnt aroma, and the rich 3-methyl-1-butanol in *C. paliurus*–kiwi wine is related to the action of the BV818 yeast strain. Moreover, phenylethyl alcohol is converted from phenylalanine and has a pleasant floral and honey fragrance. The *C. paliurus*–kiwi wine contained two phenolics (2,4-di-tertbutylphenol and phenol and 2-methoxy-3-(2-propenyl)-) but in low amounts. Moreover, terpenoids are typical compounds in wines and directly reflect the fruit flavor profile of the wine [38]. Terpenoids were more abundant and diverse in *C. paliurus*–kiwi wine, and a few types were found in K1 and K2.

A heat map was made based on the number of volatile flavor components detected to visualize the concentration difference in flavor compounds between the *C. paliurus*–kiwi wine, kiwi wine, K1, and K2, with each compound represented by a different color shade. Figure 2C shows the variabilities of the volatile aroma substances between the *C. paliurus*–kiwi wine and the other three available wines. The volatile components of *C. paliurus*–kiwi wine and kiwi wine were richer than those of K1 and K2. The sample clustering results also indicated that the *C. paliurus*–kiwi wine was more similar in quality to kiwi wine. PCA analysis in Figure 2D reveals close associations among 100 volatile flavor compounds. Phenylacetic acid ethyl ester, terpinen-4-ol, acetic acid 2-phenylethyl ester, and 1-hexanol were highly positioned on the positive sides of both PC1 and PC2. Methyl benzoate occupied the highest sum of the absolute loading values on the sides of both PC1 and PC2; thus, methyl benzoate was proven to be a key aroma compound.

### 2.3. Odor Profiles and PCA Analysis of OAVs in Four Kiwi Wines

The ratio of volatile aroma compound concentration to an odor threshold value in a food or beverage helps determine the relative importance of aroma/flavor [39]. OAV is the average concentration of an aromatic substance divided by its odor threshold. Threshold values are susceptible to small changes in the properties and structure of a substance, resulting in large changes. When 1 > OAV > 0.1, it means that the aromatic compound can increase the aroma and harmony of the wine to some extent. When OAV > 1, the substance may have a direct influence on the aroma of the wine and be identified as a characteristic aroma substance [40]. Table 1 shows the OAVs of the four kiwi wines, which were obtained using reported sensory thresholds and aroma descriptions [41,42,43].

**Figure 3:**
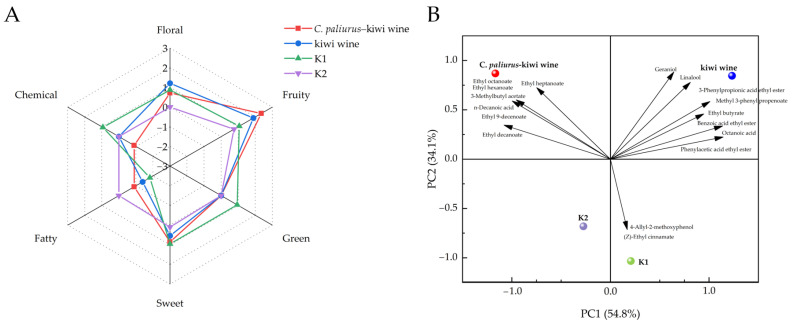
(**A**) Radar profiles of four kiwi wines. The logarithmic form of the OAVs is the coordinates of the figure. (**B**) Principal component analysis (PCA) of flavor compounds in four kiwi wine samples.

Linalool, geraniol, (Z)-ethyl cinnamate, ethyl butyrate, 3-methylbutyl acetate, ethyl hexanoate, ethyl octanoate, ethyl decanoate, benzoic acid ethyl ester, methyl 3-phenyl propenoate ethyl heptanoate, 3-phenylpropionic acid ethyl ester, and 4-allyl-2-methoxyphenol had the highest concentrations with OAVs above 1. In *C. paliurus*–kiwi wine, 3-methylbutyl acetate and ethyl heptanoate were the characteristic volatiles that differed from the other three wines and had the pronounced aromas of banana and pineapple. As shown in Figure 3A, the *C. paliurus*–kiwi wine had a more pronounced sweet and fruity aroma. LA-AU yeast can develop the complex aroma of ripe fruits, such as sour cherries, blackberries, etc., resulting in the smooth taste of the *C. paliurus*–kiwi wine. The kiwi wine had a bit more floral aroma, while K1 and K2 had a higher intensity of chemical and fatty flavor. Similarly, nine-carbon-atom straight-chain unsaturated alcohols, aldehydes, and 3-hexen-1-ol are important sources of green and fatty flavors. Zhang et al. [44] demonstrated that all three melon spirits showed a green, fatty flavor. The PCA result in Figure 3B showed that the *C. paliurus*–kiwi wine and three wines could be separated by PC1 (54.8%) and PC2 (34.1%). Ethyl hexanoate, *n*-decanoic acid, 3-methylbutyl acetate, ethyl octanoate, ethyl decanoate, ethyl 9-decenoate, and ethyl heptanoate were closer to *C. paliurus*–kiwi wine. Those volatile aroma compounds were the substances with fruity aromas, like those in bananas, green apples, and pineapples [45].

In summary, the main volatile flavor components detected in the *C. paliurus*–kiwi wine made a key contribution to the sweet aroma, delicate taste, and smooth taste with a long aftertaste of the wine, resulting in a kiwi wine with a harmonious flavor and pleasant aroma.

### 2.4. Sensory Analysis of Four Kiwi Wines

The results of the sensory analysis of wines are shown in Table 2. Among them, the *C. paliurus*–kiwi wine received the highest score, with a clear brownish-yellow color, a soft and smooth taste, and a pronounced fresh kiwifruit flavor. The total score for sensory evaluation of *C. paliurus*–kiwi wine was higher than the three kiwi wines, which meant sensory quality for *C. paliurus*–kiwi wine was improved. With the addition of *C. paliurus* aqueous extracts, the color of *C. paliurus*–kiwi wine was darker than that of kiwi wine and closer to the brown color of the kiwifruit peel. Lan et al. [46] believed that if the color of kiwi wine was similar to that of kiwifruit itself, it would be more acceptable to consumers. In conclusion, the *C. paliurus*–kiwi wine can improve the sensory quality of kiwi wine to a certain extent, and the *C. paliurus*–kiwi wine has more prospects in the future sales market.

## 3. Materials and Methods

### 3.1. Materials

Two commercial kiwi wines with high-sale volumes currently on the market were purchased and analyzed in comparison with *C. paliurus*–kiwi wine and kiwi wine. Information about the commercially available kiwi wines is presented in Table 3.

### 3.2. Preparation of C. paliurus Aqueous Extracts and Kiwifruit Pulp

Fresh “Xuxiang” kiwifruits were purchased in Yuyao City, Zhejiang Province, China. Intact mature fruits (8 °Brix) without any physical injuries with a length of 4–6 cm (substantially smaller than normal kiwifruits) were collected. Commercially dried *C. paliurus* leaves were collected in Sangzhi County, Hunan Province, China. Whole, undersized kiwifruits were washed with water and then liquefied with fruit peel to obtain a thick pulp in a blender. The leaves were ground in a blender (Zhejiang Supor Co., Ltd., Hangzhou, China) for 1 min until uniform in size, and the powder could pass through a 200-mesh sieve to make the extraction facile [47]. The ratio of *C. paliurus* powder to water was 1:50 (*w*/*v*). Extraction was performed in a water bath at 70 °C for 25 min. The *C. paliurus* aqueous extracts were added to the kiwifruit pulp after being cooled to room temperature (21 ± 3 °C). In this experiment, pectinase (>40,000 AJDU/g, Diboshi Brewing Machine Co., Ltd.) at 160 mg/L was added to the *C. paliurus*–kiwi mixed solution, and SO_2_ concentration was adjusted to 50.0–60.0 mg/L with the addition of food-grade K_2_S_2_O_5_ (each gram of K_2_S_2_O_5_ can convert to 0.520–0.570 g SO_2_) (Zhejiang Yinuo Biological Technology Co., Ltd., Wenzhou, China). The mixed solution was then placed in a 40 °C thermostat water bath for 3.5 h to obtain the *C. paliurus*–kiwi mixed enzymatic hydrolysate [48].

### 3.3. Wine Fermentation

**Scheme 1:**
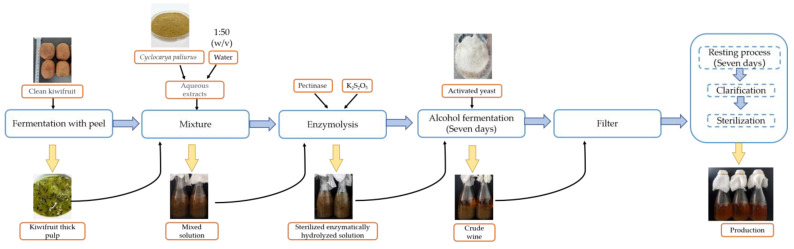
The production process of *C. paliurus*–kiwi wine.

The main fermentation was conducted for seven days and then filtrated through gauze, and then the wine was transferred to a new glass vessel to start post-fermentation for seven days (Scheme 1). The fermentation was conducted in triplicate. Wine samples were collected every two days, then sealed and stored at 4 °C until analysis.

Fermentation of *C. paliurus*–kiwi wine was conducted according to previous literature with some modifications and response surface methodology (RSM) optimization results (data provided in the supplementary material) [49,50]. The initial sugar content was adjusted to 18 °Brix with white granulated sugar (Hebei Dundao Food Technology Co., Ltd., Shijiazhuang, China). Then, *C. paliurus*–kiwi-mixed enzymatic hydrolysate was sterilized at 65 °C for 30 min and cooled to 21 ± 3 °C. The same weight of commercial Saccharomyces cerevisiae strains LA-MA, LA-AU, and BV818 were activated (hydration in a 5% glucose solution at 37 °C for 30 min) and added to the *C. paliurus*–kiwi mixed enzymatic hydrolysate. Then, the enzymatic hydrolysate was placed in the dark at 21 ± 3 °C without stirring for alcohol fermentation [51,52]. The end of alcohol fermentation was marked by the concentration of Brix keeping the same after two-day measurements. The yeast cells were removed by centrifugation at 7000 rpm for 10 min. Later, the crude *C. paliurus*–kiwi wine was filtrated through gauze and moved to a new sterilized glass bottle to rest for 7 days (Scheme 1) and clarify [53]. The resting process was carried out under the same conditions as alcohol fermentation. Citric acid and Na_2_CO_3_ were employed to adjust the pH of the fermentation broth to 3.5–4.0 throughout the *C. paliurus*–kiwi winemaking process. Kiwi wine is made solely from kiwifruit fermentation, using the same materials and methods as *C. paliurus*–kiwi wine. The fermentation was conducted in triplicate. *C. paliurus*–kiwi wine samples were collected every 2 days, sealed, and stored at 4 °C [54].

### 3.4. Nutrient and Antioxidant Capacity Analysis

The soluble solid content was measured by a hand-held Abbe refractometer (Hangzhou Luheng Biotechnology Co., Ltd., Hangzhou, China), and the results were expressed in degrees Brix. pH was determined using a pH meter (Shanghai Lichen Instrument Technology Co., Ltd., Shanghai, China). The wine was titrated to a pH of 8.2, and the total titratable acidity (TTA) content was calculated based on the conversion coefficient of malic acid [55]. Total polyphenol content (TPC) was determined by Folin–Ciocalteu colorimetry. Total flavonoid content (TFC) was determined as described previously with slight modifications to titrate with 0.1 mL/L of NaOH [56].

The antioxidant activities of the samples were evaluated using 2,2-diphenyl-1-picrylhydrazyl (DPPH; Sigma-Aldrich St. Louis, MO, USA), 2,2′-azino-bis-(3-ethylbenzothiazoline-6-sulfonic acid) diammonium salt (ABTS^+^; Sigma-Aldrich St. Louis, MO), hydroxyl (OH^−^), and superoxide anion (O_2_^−^) radical scavenging assays as described by Du et al. [57] and Panja et al. [58] with some modifications. Briefly, the DPPH radical scavenging rate was determined by the subsequent method. A total of 2 mL aqueous solution containing 0.2 mL *C. paliurus*–kiwi wine was added to 2 mL 0.1 mmol/L solution of DPPH in methanol. The ABTS^+^ scavenging rate was measured with the addition of a 0.2 mL wine sample to 2 mL of a diluted ABTS^+^ solution. OH^−^ radical scavenging rate was assayed by mixing 9 mmol/L salicylic acid/ethanol solution, wine sample, and 9 mmol/L FeSO_4_ solution with 1 mL each. A total of 4.5 mL of 50 mmol/L Tris-HCl buffer (pH 8.2) was mixed with 1 mL wine sample, and 0.4 mL of 25 mmol/L pyrogallol solution was used to determine O_2_^−^ scavenging activity.

**Figure 4:**
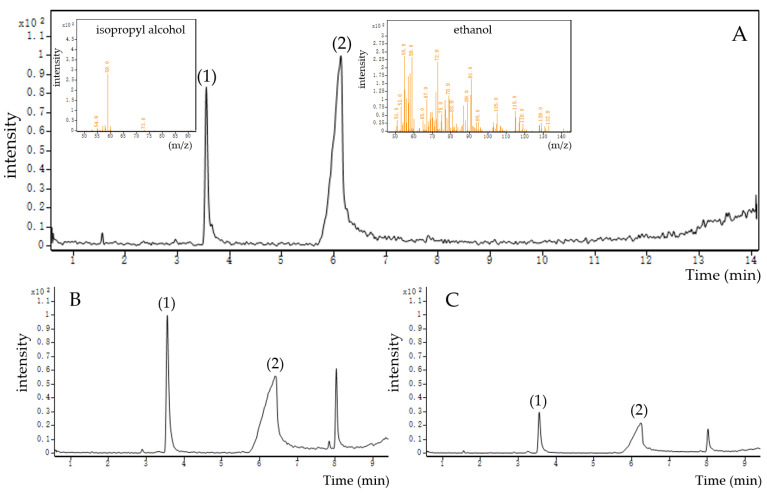
(**A**) The gas chromatogram of the standard has a 7% ethanol content. (**B**) The gas chromatograms of *C. paliurus*–kiwi wine at the end of alcoholic fermentation. (**C**) The gas chromatograms of *C. paliurus*–kiwi wine at the end of the resting process. (1) isopropyl alcohol; (2) ethanol.

Alcohol by volume (ABV) was determined based on GC–MS [59,60]. Ethanol and pure water were mixed at 2%, 3%, 4%, 5%, and 7% (*v*:*v*) to prepare the ethanol standard working fluid. The concentration of the internal standard isopropanol solution was 0.002 mL/mL. The oven temperature program started at a temperature of 40 °C, which was held for 3.8 min, and then increased to 200 °C at a rate of 15 °C/min. Finally, the temperature was kept at 200 °C for 4 min post-run. The working curve was drawn with ethanol concentration as abscissa and the ratio of the peak area of ethanol to the internal standard as ordinate. Figure 4 shows the gas chromatograms of standards and *C. paliurus*–kiwi wine samples, and the working curve was Y = −17.232X + 4.9836, R^2^ = 0.9957. The *C. paliurus*–kiwi wine sample was diluted fivefold for GC–MS analysis. The results were expressed as the arithmetic mean of three independent measurements obtained under repeatability conditions.

### 3.5. Headspace Solid Phase Microextraction/Gas Chromatography–Mass Spectrometer (HS-SPME/GC–MS) Analysis

Kiwi wine (5 mL) was pipetted into a cleaned 20 mL vial with 1 g NaCl. 2-octanol dissolved in ethanol (0.45 mg/mL) was used for semi-quantification of the aroma compounds contained in the four kiwi wines. Volatile substances were analyzed on an 8890 N gas chromatograph equipped with a 5977-mass spectrometer (Agilent Technologies, Santa Clara, CA, USA). An Agilent PAL 3 autosampler was used in the experiment. A 65/10 µm polydimethylsiloxane–divinylbenzene SPME was chosen for headspace sampling. The SPME fiber was aged for 10 min at 250 °C before each use. After the sample was balanced at 40 °C for 15 min, the extraction head was inserted into a vial to extract the sample. The extraction time was 30 min, and the agitation speed was 300 rpm. Volatile substances were collected for analysis.

The following GC–MS parameters were used: capillary column, HP-INNOWAX (30 m × 0.25 mm, 0.25 μm); injector temperature, 250 °C; ion source temperature, 230 °C; quadrupole temperature, 230 °C. The elution program started at a temperature of 40 °C, which was held for 3 min and then increased to 120 °C at a rate of 4 °C/min. Finally, the temperature was increased to 240 °C at a rate of 6 °C/min and held for 12 min. The carrier gas was ultrahigh-purity helium (99.999%) with a split injection port.

Qualitative and quantitative characterization of volatile flavor components: Identification of the compounds was achieved by comparing their mass spectra and retention indices with those available in GC–MS libraries (NIST14) and the Compound Database of Plant and Food Research, or those published in the literature (NIST Chemistry WebBook, http://webbook.nist.gov/chemistry/, accessed on 12 May 2022). Volatile compounds were semi-quantified using the ratio of their peak area to that of the 2-octanol internal standard.

### 3.6. Sensory Analysis

Sensory analysis of four kiwi wines was implemented by 15 trained panelists according to four factors, including color appearance (25.0 points), aroma intensity (30.0 points), gustatory quality and intensity (30.0 points), and typicity in the tasting stage (15.0 points). Each kind of wine sample was measured in triplicate, and panelists’ palates were requested to be cleansed thoroughly with spring water before each measurement. This study was reviewed and approved by the Zhejiang Ocean University IRB (Zhoushan, China), and informed consent was obtained from each subject prior to their participation in the study.

### 3.7. Statistical Analysis

Each glass bottle contains about 350 mL of *C. paliurus*–kiwi wine. During the winemaking process of *C. paliurus*–kiwi wine, the wine sample was measured every two days, and every sample was only used once. For the measurement of volatile aromas, three bottles of each fruit wine were used. Statistical analysis was performed using Microsoft Excel and SPSS 22.0. The detected volatiles were determined by mass spectrometry and verified by the spectral libraries (NIST 14) and their Kováts retention indices (RI). Flavor compounds with an odor activity value (OAV) > 0.1 were selected for principal component analysis (PCA). The concentration of volatile substances was normalized using log2 values and shown by a heatmap. PCA and cluster analysis were performed using Origin Pro 2022 and TBtools 1.098, respectively. Experimental data were represented by the mean ± standard deviation.

## 4. Conclusions

In this work, we analyzed the changes in physicochemical properties of *C. paliurus*–kiwi wine and compared antioxidant capacity, aroma, and sensory differences between the *C. paliurus*–kiwi wine, kiwi wine, and two commercially available kiwi wines. The results indicated that antioxidant activity was maintained at a relatively high and stable level during 14 days of the *C. paliurus*–kiwi winemaking process. TFC increased during the winemaking process. TPC increased during early fermentation and later stabilized. O_2_^−^ scavenging activity, TFC, and TPC of *C. paliurus*–kiwi wine were significantly higher than those of the other three kiwi wines. Kiwi wine had a more floral aroma, whereas K1 and K2 had a higher intensity of chemical and fatty flavor. However, there were forty-three volatile aroma substances in the *C. paliurus*–kiwi wine, which showed a stronger fruity and sweet aroma compared with commercially available wines. Ethyl hexanoate, 3-methylbutyl acetate, ethyl octanoate, ethyl decanoate, etc. were the main aroma components in this wine. The total score for sensory evaluation of *C. paliurus*–kiwi wine was also higher than that of the other three kiwi wines. The wine made from defective kiwifruit and *C. paliurus* had a distinctly fruity and sweet aroma, which made it more acceptable to consumers and had great market potential.

## Figures and Tables

**Table 1:** Aroma series and OAVs of volatile aroma substances in four kiwi wines.

Compounds	OT (μg/L)	Aroma Description	Aroma Series	OAVs			
				*C.paliurus*–Kiwi Wine	Kiwi Wine	K1	K2
Octanoic acid	500	Fatty, rancid	Fatty	0.494	0.0410	0.0150	n.d.
n-Decanoic acid	1.00 × 10^3^	Waxy, tallowy, rancid, soapy	Fatty, chemical	0.127	n.d.	n.d.	n.d.
Linalool	25.0	Citrus, floral, sweet, grape	Floral, fruity, sweet	0.708	2.79	n.d.	n.d.
Geraniol	7.50	Citric, geranium	Floral	0.877	2.09	n.d.	n.d.
(Z)-Ethyl cinnamate	1.00	Sweet, spicy, cinnamon, woody	Sweet, green, chemical	n.d	n.d.	8.63	n.d.
Ethyl butyrate	20.0	Papaya, butter, sweetish, apple	Fruity	n.d	52.7	n.d.	n.d.
3-Methylbutyl acetate	30.0	Banana	Fruity	3.53	n.d.	n.d.	n.d.
Ethyl hexanoate	8.00	Green apple, brandy, wine-like	Fruity	55.0	16.5	11.0	2.74
Ethyl octanoate	250	Sweet, fruity, pear	Fruity, sweet	6.46	0.702	0.494	1.25
Ethyl decanoate	500	Fruity, Strawberry	Fruity	2.54	0.160	n.d.	1.65
Benzoic acid ethyl ester	53.0	Rose	Floral	3.66	11.4	6.24	n.d.
Ethyl 9-decenoate	100	-	-	0.215	n.d.	n.d.	n.d.
Phenylacetic acid ethyl ester	70.0	Fruity, rose, honey	Fruity, floral	n.d	0.116	0.0600	n.d.
Methyl 3-phenyl propenoate	11.0	Cherry	Fruity	n.d	4.23	n.d.	n.d.
Ethyl heptanoate	0.170	Pineapple	Fruity	156	n.d.	n.d.	n.d.
3-Phenylpropionic acid ethyl ester	1.60	-	-	n.d	10.8	n.d.	n.d.
4-Allyl-2-methoxyphenol	5.00	Clove, honey, spice	Floral	n.d	n.d.	1.20	n.d.

Odor thresholds (OT), not detected (n.d.).

**Table 2:** Sensory analysis of four kiwi wines.

	Color Appearance (25.0 Points)	Aroma (30.0 Points)	Taste (30.0 Points)	Typicality (15.0 Points)	Total Score (100.0 Points)
*C. paliurus*–kiwi wine	22.0	22.8	23.8	9.80	78.5
kiwi wine	20.0	23.8	23.8	10.2	77.8
K1	19.5	20.5	22.0	9.50	71.5
K2	20.0	20.0	22.0	9.10	71.1

The mean scores of 15 judges (three replicates) were kept as three significant numbers.

**Table 3:** Information about the commercially available kiwi wines.

Name	Brand	Features	ABV (Vol.%)
K1	Dujiangyan Qingcheng Mountain Avenue Industrial Co. (Chengdu, China)	Fermented wine	12.5
K2	Anhui Rose Supreme Organic Food Co. (Lu’An, China)	Fermented wine	11.0

Alcohol by volume (ABV).

## Data Availability

The data presented in this study are available in the article and Supplementary Material.

## Supplementary Materials

The following supporting information can be downloaded at: https://www.mdpi.com/article/10.3390/molecules29010032/s1, Table S1: Box–Behnken design and response values for the *C. paliurus*–kiwi wine; Table S2: Variance analysis of the regression model; Figure S1: Response surface results for TFC and TTA; Table S3: Abbreviations and concentration of volatile aroma substances in Figure 2C.
