# Supplementary material for: Application of Cyclocarya paliurus–Kiwifruit Composite Fermented to Enhance Antioxidant Capacity, Flavor, and Sensory Characteristics of Kiwi Wine
Source: Molecules. 2023 Dec 20;29(1):32. doi: 10.3390/molecules29010032 (PMC10780096; doi:10.3390/molecules29010032)
Supplement: Supplementary file 1 [file molecules-29-00032-s001.zip › molecules-2740038-supplementary.pdf]

Supplementary Table S1. Box–Behnken design and response values for the *C. paliurus*-kiwi wine.

Run	Independent variable			Response	
	A Ratio (v/v)	B Yeast addition (g/L)	C Initial sugar (°Brix)	TFC (mg/100 mL)	TTA (g/L)
1	1 (1:4)	-1 (0.2)	0 (20)	12.45	23.52
2	0 (1:3)	-1	-1 (18)	10.70	25.13
3	1	-1	0	12.71	24.00
4	0	-1	1 (22)	11.95	25.35
5	-1 (1:2)	0 (0.3)	1	12.90	25.20
6	0	1(0.4)	-1	12.96	24.97
7	0	0	0	9.93	25.65
8	0	1	1	11.38	26.55
9	0	0	0	9.41	25.97
10	-1	1	0	12.27	23.97
11	1	0	-1	12.55	25.80
12	1	0	1	12.36	26.09
13	1	1	0	12.11	28.59
14	0	0	0	8.82	26.13
15	-1	0	-1	12.36	25.24

Total flavonoid content (TFC), total titratable acidity (TTA).

Supplementary Table S2. Variance analysis of the regression model.

Response value	Y1 TFC				
**Item**	**Sum of squares**	**Degree of freedom**	**Mean square**	**F value**	**P value**
Model	23.27	9	2.59	10.73**	0.0088
Residual	1.2	5	0.24		
Lack of fit	0.56	2	0.28	1.29	0.3945
Pure error	0.65	3	0.22		
R^2	0.9508				
**Response value**	**Y2 TTA**				
**Item**	**Sum of squares**	**Degree of freedom**	**Mean square**	**F value**	**P value**
Model	19.79	6	3.3	25.62**	< 0.0001
Residual	1.03	8	0.13		
Lack of fit	0.79	5	0.16	2.03	0.2972
Pure error	0.23	3	0.078		
R^2	0.9505				

*Implies a significant difference (P<0.05), **implies an extremely significant difference (P<0.01).

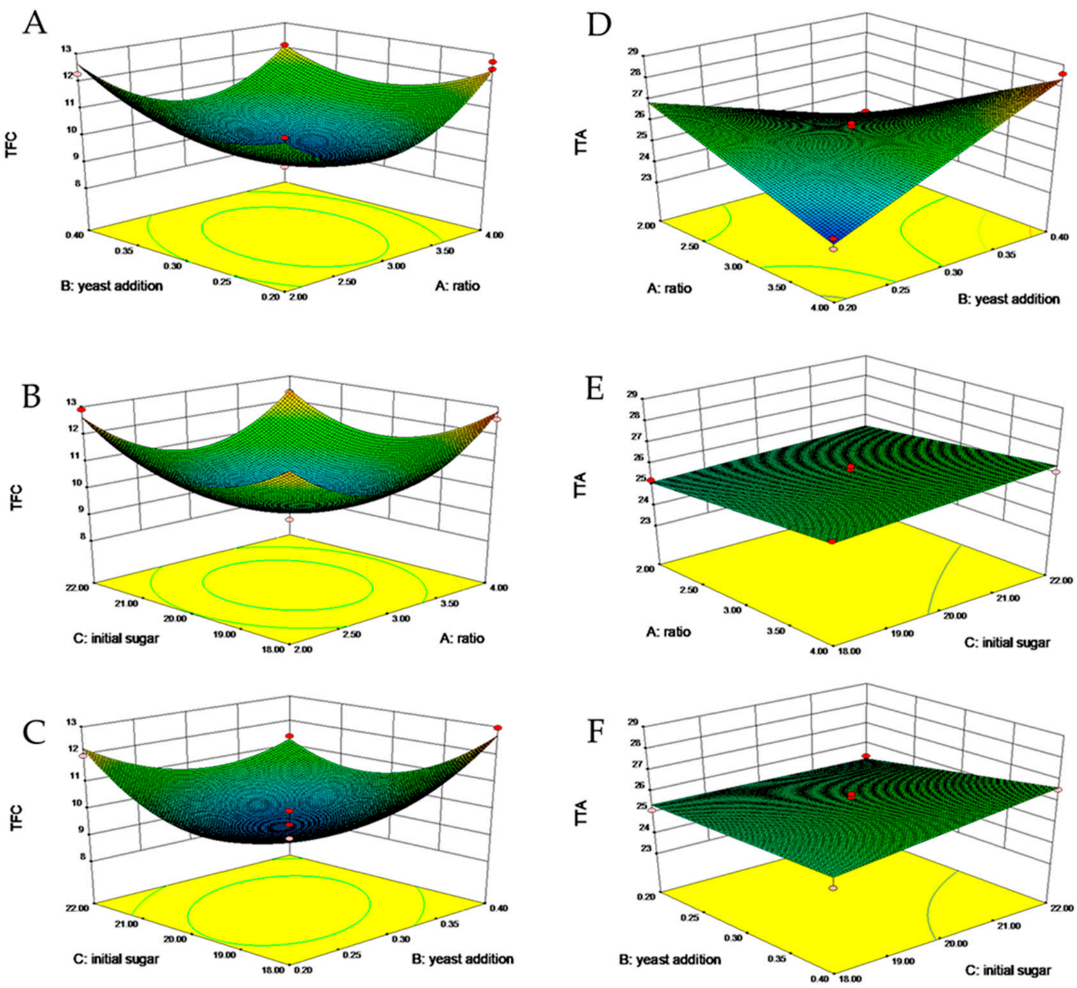

Supplementary Figure S1. Response surface results for TFC and TTA.

Supplementary Table S3. Abbreviations and concentration of volatile aroma substances in Fig. 3C.

abbreviation	Volatile	CAS	C. paliurus-kiwi wine (ug/L)	Kiwi wine (ug/L)	K1 (ug/L)	K2 (ug/L)

Ac1	Hexanoic acid	142-62-1	0.000	17.426	0.000	0.000
Ac2	Acetic acid	64-19-7	43.532	213.928	0.000	41.267
Ac3	Octanoic acid	124-07-2	247.176	20.324	7.597	0.000
Ac4	n-Decanoic acid	334-48-5	127.110	0.000	0.000	0.000
Ac5	Butyric acid	107-92-6	0.000	30.761	0.000	0.000
Ac6	Benzoic acid	65-85-0	0.000	0.000	1048.896	0.000
Ac7	Hexa-2, 4-dienoic acid	110-44-1	0.000	609.073	0.000	0.000
Ac8	2, 6-Pyridinedicarboxylic acid	499-83-2	0.000	11.365	0.000	0.000
Ac9	2-Methylhexanoic acid	4536-23-6	18.158	0.000	0.000	0.000
Ac10	Isobutyric acid	79-31-2	46.957	0.000	0.000	0.000
T1	alpha-Terpineol	98-55-5	0.000	17.053	28.023	0.000
T2	3-Cyclohexen-1-ol, 4-methyl-1-(1-methylethyl)-, (1R)-	20126-76-5	104.605	0.000	0.000	99.537
T3	Linalool	78-70-6	17.696	69.709	0.000	0.000
T4	(-)-alpha-Terpineol	10482-56-1	12.302	0.000	0.000	0.000
T5	Terpinen-4-ol	562-74-3	0.000	30.119	8.750	0.000
T6	g-Terpinene	99-85-4	0.000	0.000	0.000	61.040

T7	Geraniol	106-24-1	6.581	15.683	0.000	0.000
T8	Espatulenol	6750-60-3	12.140	0.000	0.000	0.000
Es1	Acetic acid, 2-chloro-, nonyl ester	5451-96-7	0.000	0.000	12.993	0.000
Es2	(E)-3-Hexenyl butanoate	53398-84-8	0.000	30.217	0.000	0.000
Es3	7-Octenoic acid ethyl ester	35194-38-8	0.000	0.000	11.802	0.000
Es4	3-Hexen-1-ol, 1-acetate, (3E)-	3681-82-1	0.000	17.747	0.000	0.000
Es5	Ethyl 6-heptenoate	25118-23-4	0.000	0.000	17.478	0.000
Es6	Butyl isobutyl phthalate	17851-53-5	0.000	45.035	90.423	0.000
Es7	2-Hexenoic acid, ethyl ester	1552-67-6	14.244	0.000	0.000	0.000
Es8	(Z)-Ethyl cinnamate	4192-77-2	0.000	0.000	8.632	0.000
Es9	Pentanoic acid, 2-hydroxy-4-methyl-, ethyl ester	10348-47-7	0.000	55.422	0.000	0.000

Es10	Ethyl malate	626-11-9	0.000	0.000	6.276	0.000
Es11	3, 3, 5-Trimethylcyclohexyl salicylate	118-56-9	0.000	7.051	0.000	0.000
Es12	9, 12, 15-Octadecatrienoic acid, ethyl ester, (Z, Z, Z)-	1191-41-9	7.903	0.000	0.000	0.000
Es13	Tetradecanoic acid, ethyl ester	124-06-1	15.184	0.000	6.607	10.844
Es14	(E)-4-Heptenoic acid ethyl ester	54340-70- 4	11.658	0.000	0.000	0.000
Es15	Ethyl 9-hexadecenoate	54546-22-4	20.665	0.000	0.000	11.199
Es16	Diethyl succinate	123-25-1	0.000	32.135	5.415	32.605
Es17	Ethyl butyrate	105-54-4	0.000	1054.841	0.000	0.000
Es18	Ethyl acetate	141-78-6	0.000	459.050	0.000	0.000
Es19	3-Methylbutyl acetate	123-92-2	106.034	0.000	0.000	0.000
Es20	Ethyl hexanoate	123-66-0	440.015	132.129	87.723	21.928
Es21	Acetic acid benzyl ester	140-11-4	0.000	37.602	0.000	0.000
Es22	Ethyl octanoate	106-32-1	1614.827	175.469	123.603	313.418
Es23	Ethyl nonanoate	123-29-5	21.621	0.000	15.040	0.000

Es24	Methyl benzoate	93-58-3	20.870	65.312	0.000	0.000
Es25	Ethyl decanoate	110-38-3	1272.027	79.994	0.000	826.366
Es26	Benzoic acid ethyl ester	93-89-0	193.706	602.101	330.743	0.000
Es27	Ethyl 9-decenoate	67233-91-4	21.498	0.000	0.000	0.000
Es28	Methyl salicylate	119-36-8	22.598	0.000	0.000	0.000
Es29	Acetic acid 2-phenylethyl ester	103-45-7	43.282	26.227	0.000	5.805
Es30	Ethyl dodecanoate	106-33-2	228.126	0.000	0.000	152.080
Es31	Ethyl oleate	6512-99-8	0.000	0.000	0.000	15.054
Es32	Ethyl hexadecanoate	628-97-7	59.202	20.130	36.475	105.355
Es33	Phenylacetic acid ethyl ester	101-97-3	0.000	8.115	4.197	0.000
Es34	9, 12-Octadecadienoic acid (Z, Z)-, ethyl ester	544-35-4	0.000	0.000	0.000	17.433
Es35	Methyl 3-phenyl propenoate	103-26-4	0.000	46.576	0.000	0.000
Es36	Ethyl heptanoate	106-30-9	26.507	0.000	0.000	0.000
Es37	Methyl n-caprate	110-42-9	0.000	0.000	0.000	7.004
Es38	Benzyl butyrate	103-37-7	0.000	10.929	15.567	0.000

Es39	3-Phenylpropionic acid ethyl ester	2021-28-5	0.000	17.220	0.000	0.000
Es40	Octanoic acid, 3-methylbutyl ester	2035-99-6	0.000	0.000	0.000	22.802
Es41	Ethyl 3-hexenoate	2396-83-0	37.858	0.000	0.000	0.000
Es42	Ethyl 2-furoate	614-99-3	0.000	0.000	12.536	0.000
Es43	Isopentyl hexanoate	2198-61-0	16.003	0.000	0.000	0.000
Es44	(E, E)-Ethyl2, 4-hexadienoate	2396-84-1	0.000	202.135	0.000	0.000
Es45	Formic acid, octyl ester	112-32-3	0.000	0.000	0.000	7.778
Es46	Menthyl acetate	89-48-5	0.000	111.448	0.000	0.000
Al1	7-Tetradecen-1-ol, (7E)-	37011-95- 3	0.000	0.000	7.235	0.000
Al2	trans-nerolidol	40716-66-3	0.000	5.408	0.000	0.000
Al3	3-Methyl-1-butanol	123-51-3	1323.320	176.047	0.000	725.705
Al4	1-Hexanol	111-27-3	56.552	72.399	0.000	28.394
Al5	(R, R)-2, 3-Butanediol	24347-58-8	28.559	0.000	0.000	0.000
Al6	Benzyl alcohol	100-51-6	31.923	16.916	0.000	0.000

Al7	Phenylethyl Alcohol	60-12-8	572.116	181.042	0.000	84.450
Al8	2-Methyl-1-propanol	78-83-1	0.000	0.000	0.000	206.058
Al9	1-Decanol	112-30-1	0.000	0.000	0.000	8.461
P1	2, 4-Di-tertbutylphenol	96-76-4	8.780	37.449	10.248	0.000
P2	4-Allyl-2-methoxyphenol	97-53-0	0.000	0.000	5.998	0.000
P3	2, 4, 6-Tri-tert-butylphenol	732-26-3	0.000	13.098	0.000	0.000
P4	Dihydroeugenol	2785-87-7	0.000	7.685	0.000	0.000
P5	Phenol, 2-methoxy-3-(2-propenyl)-	1941-12-4	5.238	0.000	0.000	0.000
C1	3-Hydroxy-2-butanone	514-95-4	0.000	17.064	0.000	0.000
C2	(E)-6, 10-Dimethylundeca-5, 9-dien-2-one	10208-80-7	13.331	0.000	0.000	0.000
C3	Beta-ionone	464-17-5	0.000	0.000	8.787	0.000
C4	2-Butanone, 4-(2, 6, 6-trimethyl-1-cyclohexen-1-yl)-	294-62-2	0.000	0.000	8.514	0.000
C5	Geranylacetone	99-87-6	0.000	0.000	10.151	0.000
C6	2-Oxetanone	193695-	0.000	78.754	0.000	0.000

		14-6				
H1	1, 5, 5-Trimethyl-6-methylenecyclohexene	514-95-4	15.213	0.000	0.000	0.000
H2	α-muurolene	10208-80-7	0.000	0.000	0.000	23.614
H3	1, 7, 7-Trimethyl-2-norbornene	464-17-5	0.000	58.902	0.000	0.000
H4	Cyclododecane	294-62-2	0.000	0.000	4.609	0.000
H5	4-Isopropyl-1-methylbenzene	99-87-6	0.000	0.000	0.000	23.627
H6	Bicyclo [3. 2. 1] octane, 7-ethylidene-1, 2, 8, 8-tetramethyl-, (1R, 2R, 5R, 7E)-	193695-14-6	0.000	0.000	28.531	0.000
O1	Methoxyphenyloxim	1000222-86-6	0.000	12.973	10.660	14.798
O2	1, 4-Bis(trimethylsilyl)benzene	13183-70- 5	0.000	0.000	8.103	0.000
O3	Cyclooctasiloxane,hexadecamethyl-(7CI, 8CI, 9CI)	556-68-3	0.000	18.693	0.000	0.000

O4	Decamethylcyclopenta siloxane	541-02-6	115.197	162.196	117.408	0.000
O5	Hexamethylcyclotrisil oxane	541-05-9	0.000	0.000	0.000	6.551
O6	Octamethylcyclotetrasi loxane	556-67-2	0.000	0.000	7.256	0.000
O7	Dodecamethylcyclohe xasiloxane	540-97-6	147.167	306.362	176.457	51.803
O8	Dimethoxydimethylsil ane	1112-39-6	15.259	0.000	0.000	75.827
O9	Silanediol, 1, 1-dimethyl-	1066-42-8	0.000	0.000	35.161	34.592
Et1	Dimethyl ether	115-10-6	1986.189	3346.161	3321.422	4125.634
